# Past, Present and Future of Surgical Meshes: A Review

**DOI:** 10.3390/membranes7030047

**Published:** 2017-08-22

**Authors:** Karen Baylón, Perla Rodríguez-Camarillo, Alex Elías-Zúñiga, Jose Antonio Díaz-Elizondo, Robert Gilkerson, Karen Lozano

**Affiliations:** 1 Centro de Innovación en Diseño y Tecnología, Tecnológico de Monterrey, Campus Monterrey, Monterrey 64849, Mexico; karen.baylon@itesm.mx (K.B.); A01139114@itesm.mx (P.R.-C.); aelias@itesm.mx (A.E.-Z.); 2 Escuela de Medicina, Tecnológico de Monterrey, Campus Monterrey, Monterrey 64849, Mexico; jadiaze@itesm.mx; 3 Departments of Biology and Clinical Laboratory Sciences, The University of Texas Rio Grande Valley, Edinburg, TX 78539, USA; robert.gilkerson@utrgv.edu; 4 Mechanical Engineering Department, The University of Texas Rio Grande Valley, Edinburg, TX 78539, USA

**Keywords:** surgical mesh, hernia repair, abdominal wall reconstruction, biocompatibility

## Abstract

Surgical meshes, in particular those used to repair hernias, have been in use since 1891. Since then, research in the area has expanded, given the vast number of post-surgery complications such as infection, fibrosis, adhesions, mesh rejection, and hernia recurrence. Researchers have focused on the analysis and implementation of a wide range of materials: meshes with different fiber size and porosity, a variety of manufacturing methods, and certainly a variety of surgical and implantation procedures. Currently, surface modification methods and development of nanofiber based systems are actively being explored as areas of opportunity to retain material strength and increase biocompatibility of available meshes. This review summarizes the history of surgical meshes and presents an overview of commercial surgical meshes, their properties, manufacturing methods, and observed biological response, as well as the requirements for an ideal surgical mesh and potential manufacturing methods.

## 1. Introduction

A hernia is defined as a protrusion or projection (prolapse) of an organ through the wall of the cavity where it is normally contained [1]. There are many types of hernia, mostly classified according to the physical location, with the abdominal wall being the most susceptible site. Specifically, reports show that the most frequently seen hernia is the inguinal hernia (70–75% of cases), followed by femoral (6–17%) and umbilical (3–8.5%) hernias [2]. Hernias are also found in other sites such as the ventral or epigastric hernia, located between the chest cavity and the umbilicus.

Hernias can be uncomfortable and are sometimes accompanied by severe pain, which worsens during bowel movements, urination, heavy lifting, or straining [3]. Occasionally, a hernia can become strangulated, which occurs when the protruding tissue swells and becomes incarcerated. Strangulation will interrupt blood supply and can lead to infection, necrosis, and potentially life-threatening conditions [4].

Hernia repair is one of the most common surgical procedures performed globally. It is estimated that there are over 20 million hernia repair procedures per year worldwide [5]. The number of procedures has been increasing and is predicted to further increase due to several risk factors such as obesity and prior abdominal surgeries [6]. Hernia repairs provide an important revenue stream for hospitals, estimated at $48 billion/year in the United States [7].

The use of hernia mesh products to surgically repair or reconstruct anatomical defects has been widely adopted: in fact, more than 80% of hernia repairs performed in United Sates use mesh products [8]. The surgical mesh firmly reinforces the weakened area and provides tension-free repair that facilitates the incorporation of fibrocollagenous tissue [9]. However, there are many types of meshes and there is a strong controversy regarding optimum performance and success of surgical procedures. Researchers have investigated metals, composites, polymers and biodegradable biomaterials in their quest to attain the ideal surgical mesh and implantation procedure [10]. The sought-after characteristics are inertness, resistance to infection, the ability to maintain adequate long-term tensile strength to prevent early recurrence, rapid incorporation into the host tissue, adequate flexibility to avoid fragmentation, non-carcinogenic response and the capability to maintain or restore the natural respiratory movements of the abdominal wall [9].

Currently, utilized surgical meshes exhibit many but not all of the desired characteristics [8]. Therefore, current research efforts focus on providing potential solutions that range from the utilization of novel materials to new designs that could ameliorate existent shortcomings [11]. The aim of this review is to illustrate the current research in surgical meshes used for hernia repair. This review provides a perspective of existent commercial surgical meshes, their properties, manufacturing procedures, and observed biological responses. Furthermore, the article seeks to establish the requirements for an ideal surgical mesh and potential manufacturing procedures.

## 2. History

In 1890, Theodor Billroth suggested that the ideal way to repair hernias was to use a prosthetic material to close the hernia defect [12]. Many materials were used, but all failed due to infections, rejections, and recurrences [13]. Surgeons concluded that the main problem was built upon the multifilament suture material, which has been proven unsuitable in many other surgical procedures [14]. Surgeons became disenchanted with the popular cotton and silk sutures because of the frequently observed rejection syndrome and resultant endless recurring infections. The use of such sutures to secure mesh in place undoubtedly contributed to aggravate the existing bias against the surgical meshes [15].

In 1955, Dr. Francis Usher focused his attention on the materials that could solve existing problems. Nylon, Orlon, Dacron and Teflon were studied and were observed to have a variety of shortcomings such as: foreign body reaction, sepsis, rigidity, fragmentation, loss of tensile strength and encapsulation [16]. All of these precluded the acceptance of polymeric materials. After reading an article about a new polyolefin material (Marlex), which demonstrated remarkable properties, Usher started to develop a woven mesh [17]. Two years later, the marlex prostheses were implemented. These were made of large pores, which facilitated incorporation despite infections. The growth of tissue through its interstices was the main difference when compared to previous materials. After a few days of surgical incorporation, fibroblast activity was noticed to increase, more collagen was induced without giant cells, and the whole system gained strength [18]. Despite the numerous advantages of the woven and knitted polyethylene mesh, Usher continued the search for better systems. He soon found that knitted polypropylene had many more advantages: it could be autoclaved, had firm borders coupled with two-way stretching, and could be rapidly incorporated. Finally, in 1958, Usher published his surgical technique using a polypropylene mesh, and 30 years later the Lichtenstein repair (known today as “tension-free” mesh technique) was popularized for hernia repair [18]. Even when the benefits of meshes were accepted, the recollection of evidence-based cases was required to statistically quantify their advantages. In 2002, the European Union Hernia Trialists Collaboration, a group of surgical trialists who have participated in randomized trials of open mesh or laparoscopic groin hernia repair, analyzed 58 randomized controlled trials and concluded that the use of surgical meshes was superior to other techniques [19]. In particular, they noted fewer recurrences and less postoperative pain with mesh repair. These results were supported by other studies that demonstrated that hernia repair using surgical meshes reduced the risk of hernia recurrence compared to hernia reconstruction through other methods, in 2.7% vs. 8.2% in ventral hernia repair cases and by 50–75% of improvement through surgical meshes in inguinal repair [8].

Today, many surgeons agree that use of a prosthetic mesh is the preferred way to repair hernias. It should be emphasized that in the past, the success of repair was evaluated based on the strength and permanency of the mesh itself, not on the degree of scar tissue or other factors, which subsequently develop in and around the mesh [20]. The biocompatibility of the material has proven to be a strong contributor in the rejection of the prosthesis due to scar tissue developed by the immunological system. When a surgical mesh is implanted and lacks appropriate biocompatibility (either due to the material that it is made of or its structural design) the body responds by encapsulating the foreign system leading to the formation of a stiff scar which consequently results in poor tissue incorporation, causing hernia recurrence or infection of the mesh. A large percentage of meshes then have to be removed: approximately 69% of the explanted meshes are due to prosthesis infection [21].

Although the only treatment is surgery, there are new surgical procedures that ameliorate postoperative side effects such as the laparoscopic approach. Open surgery repair is performed by making an incision in the abdomen to identify and dissect the hernia sac through the subcutaneous tissues and fascia. Once the hernia sac is dissected away from any adjacent structures and examined for contents (intestine or any other tissues), these are inserted back into the peritoneal space, and hernia repair is carried out. Repair can be executed in two ways: (1) primary repair and (2) patch or mesh. The first involves sewing the tissue of the abdominal wall using sutures, while the second technique relies in the placement of a mesh to cover the hernia defect and reinforce surrounding tissue, fixing it with fibrin glue, staples or sutures.

In the case of a laparoscopic procedure, the surgeon starts by making several small incisions in the abdominal wall surrounding the hernia sac, in order to introduce surgical instruments and a laparoscope. In one of the incisions, carbon dioxide gas is introduced into the abdomen. The mesh or patch is then introduced, unrolled and fixed with staples or tacks. The procedure then continues with the release of the gas from the abdomen and closure of cutaneous incisions with sutures [22].

## 3. Current Research on Surgical Meshes

Most surgical meshes used currently are chemically and physically inert, nontoxic, stable and non-immunogenic. However, none of them are biologically inert, a property related to the mesh physiology and its role into the hernia repair process [23]. Implantation of any prosthetic material is quickly followed by an extraordinarily complex series of events that mark the initiation of the healing process [14]. As for the physiology of abdominal mesh implantation, perhaps the greatest concern, and hence the area that most research focuses on, is inflammation and wound healing [24]. The passive substrate of the biomaterials in conjunction with devitalized tissues can actively contribute to bacterial growth, resulting in infection, which delays the wound healing process [25].

The introduction of a foreign material into the body triggers a healing response characterized by one of three stereotypical reactions: (1) destruction or lysis, (2) inclusion or tolerance, and (3) rejection or removal. When an implant is introduced into the body, the immune system recognizes it as a foreign material and therefore attempts to destroy it [26]; immunosuppressive drugs must be administered to prevent the body from attacking it [27]. The rejection of an implant is primarily driven by the immune response of the T lymphocytes (T cells). The T cells are stimulated by the presence of an antigenic determinant on the foreign material. T cells are reproduced faster than the time required for immunosuppressants to interfere with its proliferation, therefore resulting in rejection of the implant given the large number of T cells attacking the foreign material [28].

Inflammation is the reaction of vascularized living tissue to injury and is the primary biological reaction to implanted medical devices. In the case of implanted meshes, the inflammatory response is presented in four stages that are related both temporally and hierarchically [29]. Immediately after implantation, prosthetics adsorb proteins, which create a coagulum around it [30]. Coagulums are composed of albumin, fibrinogen, plasminogen, complement factors and immunoglobulins [31]. Platelets adhere to the proteins releasing a host of chemoattractants that invite other cells such as polymorphonucleocytes (PMNs), fibroblasts, smooth muscle cells and macrophages to the area in a different sequence [32]. The chemotaxis process is defined as the movement of cells towards a preferred migration site triggered by a chemical stimulus [33]. The attraction of PMNs, also known as neutrophils, to the wound site is attributed to chemotaxis, and is observed as the first stage of biological response to the injured site. During the first stage or acute phase of inflammation, neutrophils phagocytize microorganisms. The neutrophil may also degenerate and die during this process, releasing its cytoplasmic and granular components near or over the surface of the prosthesis, which may also mediate the subsequent inflammatory response [34].

When the acute inflammatory response is unable to eliminate the injurious agent or restore injured tissue to its normal physiological state, the condition could progress into a state of chronic inflammation, known as second stage of inflammation. In this stage, monocytes that have migrated to the wound site during the acute inflammatory response rapidly differentiate into macrophages. In addition to macrophages, other primary cellular components such as plasma cells and lymphocytes actively contribute to the inflammatory process. Macrophages increasingly populate the area to consume foreign bodies as well as dead organisms and tissue [14].

In most of the cases where chronic inflammation is related to a medical device or biomaterial, the inflammation process will lead to an immune response or foreign body reaction, corresponding to the third stage of inflammation, where chronic inflammation macrophages fuse into a foreign body giant cell as a response to the presence of large foreign bodies [35]. Foreign body reaction is a complex defense reaction involving: foreign body giant cells, macrophages, fibroblast, and capillaries in varying amounts depending upon the form and topography of the implanted material [36].

The fourth stage of inflammation occurs in the wound healing phase and is characterized by the replacement of damaged tissue with various cells that specialize in secreting extracellular matrix materials to form a scar [14]. Wound healing and scar formation follow the initiation of inflammation, but their progression and the magnitude of scarring can be affected by the degree of persistent inflammatory activity as well as the severity of the primary injury [37].

Fibroblasts are cells that mediate the wound healing phase. These cells enter the wound site two to five days after the injury occurs, typically once the inflammatory phase has ended. Fibroblasts proliferate at the wound site, reaching peak levels after one to two weeks. The main function of fibroblasts is to synthesize extracellular matrix and collagen to maintain the structural integrity of connective tissues; at the end of the first week, these are the only cells in charge of collagen deposition. Cells involved in the regulation of inflammation, angiogenesis (formation of new blood vessels from preexisting vasculature) and further connective tissue reconstruction attach to, proliferate, and differentiate on the collagen matrix laid down by fibroblasts [26].

From a histological standpoint, the interaction between prosthesis and organism is characterized by three main aspects: size of tissue reaction; cell density; and fibroblastic activity. As mentioned, fibroblastic activity peaks one to two weeks post-wounding, usually on the 8th day for the intraperitoneal plane and on the 10th day for the extraperitoneal plane. The optimum quantity of fibroblasts needed for a successful integration of the mesh is achieved approximately two weeks after wounding. Further accumulation of fibroblasts will cause an inflammatory phase with increased fibrosis and faster prosthesis integration associated with paresthesia and pain. Furthermore, the inflammatory process could cause contraction and shrinkage of the mesh, resulting in adhesions and fistulas, leading to prosthesis rejection and eventually explantation [25].

The wound repair process described above creates a mesh integration due to the conformational changes of the proteins. This integration is progressive, starting from the prosthesis implantation that is accompanied by the foreign body reaction followed by the inclusion of the prosthesis, which occurs within the first two weeks. The process is finalized as the overall strength increases gradually, which last about 12 weeks and results in a relatively less elastic tissue that has only 70–80% of the strength of the native connective tissue [32].

Although integration and collagen deposition that result from the inflammatory response provide long-term strength, as pointed out, an aggressive integration could also be harmful to the tissue that surrounds the wound site causing a severe body reaction, inflammation, fibrosis, infection, and mesh rejection [23]. The fibrotic reaction generated by the body when a prosthetic material is introduced, such as in the case of surgical meshes for a hernia repair, is governed by the chemical nature of the material implanted and its physical characteristics. The integration and overall healing process of implantable surgical meshes is highly dependent upon the intrinsic mesh characteristics such as, the primary material, filament structure, tailored coatings, and pore size.

Research in abdominal wall repair has provided valuable information on the parameters, properties, and design of the meshes that influence the immune reaction of the body to the prosthesis as well as the optimal parameters to reduce fibrosis [38,39]. These factors are discussed below.

### 3.1. Elasticity and Tensile Strength

A deterioration of the tensile strength of the mesh or a strained mesh could potentially lead to hernia recurrence or a poor functional result. Hence, materials employed in surgical meshes must possess the minimum mechanical properties necessary to withstand the stresses placed on the abdominal wall. The maximum intra-abdominal pressure generated in a healthy adult occurs when coughing or jumping and is estimated to be approximately 170 mmHg. Given this information, the mesh used to repair abdominal hernias must withstand at least 180 mmHg (20 kPa) before failing [38].

The tension placed on the abdominal wall can be calculated using Laplace’s law relating the tension, pressure, thickness, and diameter of the abdominal wall. According to the thin-walled cylinder model, the total tensile strength is independent of the thickness of the layer. Hence, a physiological tensile strength of 16 N/cm is defined, using a pressure of 20 kPa (2 N/cm^2^ as the maximum pressure to be experienced in the intra-abdominal wall), and 32 cm as the longitudinal diameter of the abdominal wall [39].

Studies over human abdominal walls have demonstrated that at the maximum tensile strength of 16 N/cm, the abdominal wall in males presents a natural mean distension of 23% ± 7% and 15% ± 5% when tissue is stretched in vertical and horizontal direction, respectively. In females, a distension of 32% ± 17% and 17% ± 5% in vertical and horizontal stretching has been observed [40].

### 3.2. Pore Size

Porosity plays a key role in the reaction of the tissue to the prostheses. Bacterial growth and cell proliferation are highly dependent upon porosity and pore size. Bacterial colonies are established principally in the spaces between pores and fibers. Macroporous meshes that have large pores have shown to facilitate entry of macrophages, fibroblasts and collagen fibers that will constitute the new connective tissue, integrate the prosthesis to the organism and prevent colonization of bacteria. Large pores have shown easy infiltration of immunocompetent cells, providing protection from infection [25]. Microporous meshes, with pores of <10 µm, have shown a higher rejection rate given that scar tissue rapidly bridges small pores resulting in minimum integration, these meshes are associated with chronic inflammation.

Although it would be helpful to classify pore size in a standard form, currently, there is not a formal classification. Earl and Mark proposed the following: very large pore: >2000 µm; large pore: 1000–2000 µm; medium pore: 600–1000 µm; small pore: 100–600 µm and microporous (solid) <100 µm [32,41].

### 3.3. Weight (Density)

Prostheses can be classified as: heavy-weight (HW), when they are above 80 g/m^2^; mediumweight (MW), between 50 and 80 g/m^2^; light-weight (LW), between 35 and 50 g/m^2^; and ultra-lightweight, below 35 g/m^2^ [25]. While a heavy-weight mesh is produced with heavy materials, small pore size and high tensile strength, a light-weight is composed of thin filaments with large pores, generally larger than 1 mm. Given that light-weight meshes contain less material, results have shown that less pronounced foreign body reaction is to be expected. A decreased inflammatory response results in better tissue incorporation [42].

### 3.4. Constitution

Surgical meshes could be fabricated using monofilament or multifilament (twisted) systems. A surgical mesh formed of monofilament yarns provides satisfactory reinforcement ability, but with stiffness and limited pliability. In contrast, a surgical mesh formed of multifilament yarns is soft and pliable. However, multifilament yarns meshes tend to harbor infectious matter such as bacteria, increasing erosion rates by 20–30% [43]. Particularly, the small void areas or interstitial spaces between the multifilament yarns may promote the replication and breeding of such bacteria, which measures approximately 10 µm.

### 3.5. Material Absorption

Surgical meshes could be made from an absorbable or non-absorbable material. Non-absorbable meshes can withstand the mechanical requirements, are easy to shape intraoperative and have long-term stability. However, complications such as mesh stiffness over time, hernia recurrence, mesh erosion, and adhesions have been documented. On the other hand, absorbable meshes were developed to reduce these long-term complications. These meshes favor postoperative fibroblast activity. Nevertheless, after prosthesis absorption, the resulting scar tissue is not as strong as it was, and alone is insufficient to provide the needed strength and could result in hernia recurrence.

### 3.6. Commercially Available Surgical Meshes

The ideal mesh should be able to be held in situ by peripheral sutures, resist the possibility of loading under biaxial tension (coughing or lifting actions) without failure especially during the early postoperative period, and should promote a fast and organized response from fibrous tissue with minimal inflammation [3].

Given the difficulty to find a single surgical mesh that fulfills all of the “ideal” characteristics, there are more than 70 meshes for hernia repair available in the market. These are classified according to the composition or type of material as: (1) first generation (synthetic non-absorbable prosthesis), (2) second generation (mixed or composite prosthesis), and (3) third generation (biological prosthesis).

#### 3.6.1. First Generation Meshes

First generation surgical meshes are predominantly based on polypropylene (PP) systems. In 1958, the first polypropylene mesh was used to repair an abdominal wall; it was a heavyweight mesh with small pores. Due to intense fibrotic reactions, the search for an “ideal” mesh continued. In 1998, a lightweight first generation mesh was introduced: this system had larger pores and smaller surface area [38,43]. First generation meshes are mostly classified into three categories: (1) macroporous meshes, (2) microporous meshes, and (3) macroporous meshes with multifilament or microporous components.

Macroporous prostheses are characterized by a pore size larger than 75 µm. Polypropylene has been the material of choice with several brand names such as: Marlex, Prolene^®^, Prolite^®^, Atrium^®^ and Trelex^®^.

Microporous meshes have smaller pores, commonly less than 10 µm and commonly made from expanded polytetrafluoroethylene (e-PTFE) under the brand name Gore-Tex^®^ (AZ, USA).

Macroporous meshes with multifilament or microporous components contain plaited multifilamentary threads in their composition, the space between the threads is less than 10 µm and their pores are larger than 75 µm. Several systems are in the market such as: plaited polyester (PL) meshes (Mersilene^®^ and Parietex^®^); plaited polypropylene (SurgiPro^®^, Minneapolis, MN, USA), and perforated polytetrafluoroethylene (PTFE) (Mycromesh^®^ and MotifMesh^®^) [25]. Table 1 shows the classification of commercially available first generation surgical meshes.

#### 3.6.2. Second Generation Meshes

Despite the improvements made within the first generation meshes (Table 1), which include high tensile strength in order to support intra-abdominal pressure, several complications such as hernia recurrence, infection, and adhesions still prevailed. Therefore, second generation meshes were developed combining more than one synthetic material into their composition. Nearly all of these kinds of meshes continued to use PP, PL or e-PTFE but now in combination with each other and/or with other materials such as titanium (Ti), omega 3, poliglecaprone 25 (PGC-25) and polyvinylidene fluoride (PVDF) as composite systems.

The main advantage of these composite meshes relied in the fact that these could be employed in intraperitoneal spaces causing minimal adhesion formation to neighboring surfaces given that each side of the mesh is tailored to specific needs. These meshes therefore require a specific orientation during implantation; the visceral side has a microporous surface to prevent visceral adhesion, whereas the non-visceral side is often macroporous to allow parietal tissue ingrowth. There are two categories of composite meshes: absorbable and permanent (non-absorbable). Absorbable composite meshes require hydration prior to usage, are not amenable to modification, mitigate viscera-mesh related complications, and can aid in tissue ingrowth. Parietex^®^ is the first composite mesh to offer a resorbable collagen barrier on one side to limit visceral attachments combined with a three-dimensional polyester knit structure on the other side, to promote tissue ingrowth. Permanent composite meshes can be modified to fit specific applications and present less visceral adhesions and complications, taking advantage of the properties of both macro and micro porous meshes. Dual Mesh® (W.L. Gore & Associates, Inc., AZ, USA), Dulex^®^ and Composix^®^ (both manufactured by Bard Davol Inc., Providence, RI, USA)are some of the brand name meshes included in this category [42]. Table 2 lists some of the commercially available second generation surgical meshes. 

#### 3.6.3. Third Generation Meshes

Even with the improvements made on the second generation meshes (Table 2) where composite systems were designed to maintain the mechanical stability of first generation meshes (Table 1) and reduce inflammation and infection risk by mesh surface modification, the problems encountered with second generation meshes, such as the prevalence of adhesions, led to the development of biologic prostheses. Biologic mesh materials are based on collagen scaffolds derived from donor sources and they represent the so-called third generation meshes. Dermis from human, porcine, and fetal bovine sources are decellularized to leave only the highly organized collagen sources in addition to the dermal products included in porcine small intestine submucosa and bovine pericardium. The concept of these surgical meshes is that they provide a matrix for native cells to populate and generate connective tissue that could replace the tissue in the hernia defect [25]. Table 3 lists some of the commercially available third generation surgical meshes.

Third generation surgical meshes (Table 3) serve as biological scaffolds for repopulation and revascularization of host cells, showing a superior biocompatibility than first and second generations. These meshes do not trigger an inflammatory response from the body, though their high cost has hampered their wide acceptance.

### 3.7. Manufacturing Processes for Surgical Meshes

Surgical meshes are produced from different synthetic materials and in different mesh structures, the knitted structure being the most common [44]. Surgical filaments are mainly manufactured by extrusion processes and then knitted accordingly. As mentioned, meshes are typically manufactured from PL, PP, PTFE, e-PTFE, PVDF and composite materials (e-PTFE/PP) [45]. The knitting pattern can be significantly altered resulting in a broad range of properties. Thickness, pore size, tensile strength, flexural rigidity, and surface texture are highly dependent upon the knitting pattern; the resultant interplay among these characteristics imparts different performance [44]. These characteristics, besides altering the biocompatibility of the mesh given its affinity to cells, also dictate the mechanical properties of the mesh such as rigidity and deformation. Knitted meshes are a subset of the non-woven mesh configuration. However, there is much more order and consistency with pore size using a knitted design [46]. Knitting, by definition, is the construction of a fabric or cloth from the interlocking of threads through the formation of loops. Recent studies have been focused on treating the surgical mesh as a high-tech textile rather than as a prosthesis [44].

#### 3.7.1. The Extrusion Process

Melt extrusion is the least expensive and simplest form of fiber extrusion [47]. This process consists of melting the polymer pellets through a combination of applied heat and friction. The molten polymer is then forced under high pressure through a small orifice or a “shower head” spinneret. The molten polymer flows out of the spinneret and freezes into a solid fiber, which is then typically reheated and drawn numerous times to further align the molecules and hence strengthen the fiber [48].

Most of the surgical meshes are made from filaments initially developed to be used for surgical sutures. Surgical sutures are made from polymers like PP [49], PL [50], e-PTFE [51] or PVDF [52] monofilaments and have been successfully used by the medical profession for decades. Filaments used for surgical sutures have to possess several characteristics such as [53]:

1. Ability to attach to needles by the usual procedure.
2. Capability to be sterilized using ethylene oxide or ultraviolet radiation.
3. Ability to pass easily through tissue.
4. Ability to resist breakdown without developing an infection.
5. Possess minimal reaction with tissue.
6. Maintain its in vivo tensile strength over extended periods.

Commonly, the monofilaments used for surgical meshes have diameters in the range of 100–300 microns [54]. Multifilaments have also gained attention and have been used to fabricate surgical meshes. Lubricants are commonly applied to these filaments before the yarns are knitted. Suitable lubricants can be either hydrophobic lubricants [55] or hydrophilic lubricants such as polyalky glycol [56].

#### 3.7.2. The Knitting Process

**Figure 1:**
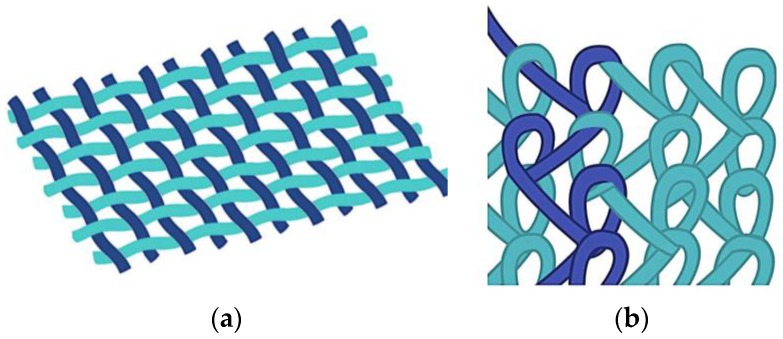
Schematic of: (**a**) woven; and (**b**) warp knitted structures.

During the knitting process, fibers or yarns are curved to follow a meandering path and not oriented unilaterally as in weaving; therefore, the resulting fabric tends to be much more flexible and elastic than woven fabrics. The basic structure of a knitted fabric consists of courses and wales. Courses are rows running across the width of the fabric, while wales are columns running across the length of the fabric. When the wales are perpendicular to the course of the fiber/yarn, this is called weft knitting. When the courses and wales are approximately parallel to the direction of the fiber/yarn, the process is known as warp knitting [57]. Figure 1 shows a warp structure.

Warp knits and weft knits have been generated for use as implantable meshes to repair specific tissue sites and organs, such as those needed in hernia repair. Because of the looped stitches, the knitted structure is soft, flexible, and stretchable. It easily adapts to the movement of the human body [58], and has high elasticity, tensile strength, bursting strength and excellent porosity, which are key requirements for any implantable device that needs to mimic the biomechanical characteristics of the abdominal wall: tension of 16 N/cm with a 38% elasticity [38]. Given the interweaving, warp-knitted materials have a fixed structure that neither loosens nor peels off during cutting, regardless of the direction [55]. These material systems have been successfully commercialized and currently used worldwide. Table 4 lists some commercially available meshes classified according to the knitted technique, material, and type of filament.

The most commonly used systems in the knitting manufacturing process are the Tricot [60] and Raschel knitting machines [61], which are used to create warp or weft knitting structures [62]. Warp knitted meshes are the most popular system used to repair hernia defects, and are manufactured using the Raschel machine with a basic configuration consisting of two bars where latch-type needles are collectively mounted (running the full knitting width of the machine) and guide bars to hold yarn beams individually. The needle bars follow up and down movements, while the guide bars move back and forth across the needles of each bar to form continuous loops. The warp knit fabric design and lapping sequence is controlled by the shagging or traverse motion of the guide bars [63].

In principle, the Tricot knitting machine is very similar to the Raschel knitting; the only difference is the use of spring beard or compound needles instead of the latch needles used in the Raschel knitting machine. In addition, Tricot sinkers not only performed the function of holding down the loops whilst the needles rise as Raschel sinkers, but also support the fabric loops. The small angle of fabric take-away and the type of knitting action in Tricots creates a gentle and lower tension on the knitted fabric, ideal for high-speed production of fine gauge [64].

A double Raschel warp knitting machine (DR 16 EEC/EAC) has 16 guide bars and enables the production of textiles with different yarn materials and counts. The machine is equipped with two different gauges, E18 and E30. This system allows the design of a mesh configuration that could be adjusted to match given design parameters such as size, shape, Young modulus, and porosity [65]. The ultimate mechanical properties of the meshes are determined by the intrinsic properties of the filaments and the final configuration of the knitted fabrics.

## 4. Future Perspectives

Despite the clinical success and vast body of knowledge that has been gained regarding manufacturing of surgical meshes, material properties, and surgical procedures, it is obvious that the ideal mesh has not been developed. It is well known that meshes still suffer from contraction and/or infection after implantation [66]. Furthermore, adhesions between the visceral side of the mesh and adjacent organs still occur. These complications may have serious consequences, such as chronic pain, intestinal obstruction, bowel erosion, or hernia recurrence. All of these problems have opened a great number of opportunities to create a new generation of surgical meshes [67]. This new generation will have to show a better integration with the tissue of the abdominal wall, but no adhesions on the visceral side. Based on the ideas of van’t Riet [68], Ebersole [69] and Xu [70], new alternatives rely broadly on surface mesh modification by novel coatings to existent meshes and/or integration of nanofiber based systems.

### 4.1. Coatings

A variety of biocompatible and biodegradable natural and synthetic polymers are being investigated. Extensive research focuses in the development of a bi-layer composite hernia mesh in order to minimize the risk of infections and reduce adhesions on the visceral side [71,72]. Materials that had been studied are: Polylactic acid (PLLA) [20], oxygenated regenerated cellulose (ORC) [67], n-vinyl pyrrolydone (NVP) and n-butylmethacrylate (BMA) [67], polyglycolic acid (PGA) [73], carboxymethylcellulose (SCMC) [74], omega-3 fatty acid [75], messenchymal stem cells (RMSC) [76], human dermal (HDF) and rat kidney fibroblasts (RKF) [76], collagen [77,78,79], chitosan [80], nanocrystalline silver particles (NCSP) [81] and titanium [82,83]. Table 5 shows some of the properties that have made these materials attractive as active ingredients in surgical meshes [71,80,84,85,86].

Most of the recently published literature still presents PP surgical meshes as the “gold standard” though with surface modifications made with materials mentioned in Table 5. Studies have primarily concentrated on: thickness and concentration of the materials used in the coating to be in contact with the visceral and/or abdominal side (Ex: 95% of oxidized collagen and 5% of chitosan) [26] and surface density (measured in g/m^2^). The following Table 6 presents a summary of the obtained results based on the inflammatory response and percentage of adhesion.

In general, the new composite meshes show highly improved performance regarding peritoneal regeneration and visceral adhesion [84]. These studies have developed composite surgical meshes with high potential for adoption. Further studies with a focus on long-term adhesion and structural performance will complement obtained results.

### 4.2. Nanofibers

Nanofiber systems made from a large variety of materials have been explored extensively in the last decade. Scaffolds for tissue regeneration are strongly deemed as a potential application of these systems [87]. Mimicking the extracellular matrix (ECM) is vital to control cell behavior, such as adhesion, proliferation, migration, and differentiation. Tissue Engineering (TE) has been extensively explored to provide answers associated with current problems encountered in the interaction of the surgical meshes with the human body. One of the challenges of TE is to mimic the natural extracellular matrix (ECM) of the abdominal wall to promote an efficient integration. Researchers are actively exploring the implementation of nanofiber systems to effectively mimic the ECM [88,89,90].

Nanofibrous structures present several advantages, such as high specific surface area for cell attachment, higher microporous structure and a 3D micro environment for cell–cell and cell–biomaterial contact, these being associated with unique physical and mechanical properties. These structures when compared with commercial surgical meshes possess higher porosity and smaller pore size. These properties make nanofiber systems suitable for biomaterials used in wound care, drug delivery, and scaffolds for tissue regeneration [20,44,91].

Scaffolds for tissue engineering must possess a porous structure able to facilitate cell migration, a balance between surface hydrophilicity and hydrophobicity for cell attachment, mechanical properties comparable to natural tissue, and biocompatibility. Studies have shown that the abovementioned characteristics are also highly influenced by average diameter of the fibers and pore size. Effective cell attachment and proliferation has been observed in fiber systems with average diameters smaller than 1 µm and average pore size of 14 µm [92]. In commercially available meshes, even when it has been shown that cells are able to proliferate in micrometer/macrometer regimes, the cells in fact have difficulty attaching and proliferating. Cells are seen around the fibers whereas, on nanofiber based meshes, the cells attach to the fibers and quickly proliferate while making strong contact with underlying nanofibers, therefore promoting interlayer growth.

The application of nanofiber systems has been hampered due to its poor mechanical properties and nanofiber availability. Most of the available studies have focused on nanofibers prepared through solution processes. The properties of the developed fibers can be controlled by different parameters such as utilized solvent, concentration of polymer, processing methods, and ambient conditions. For example, in the case of nanofibers made of polypropylene (one of the highly used polymers for commercially available surgical meshes), decahydronaphthalene (decalin) and cyclohexane have been used as preferred solvents. Polypropylene nanofibers prepared with cyclohexane exhibited a rougher surface when compared to the fibers prepared with decalin, suggesting that the surface morphology of the nanofibers depend on the boiling point of each solvent [93]. When stress–strain behaviors of the nanofibers are investigated, a tensile strength of 61.4 ± 1.5 MPa with 35.2% ± 1.7% of strain, and a Young modulus of 174.6 ± 1.7 MPa was obtained for the decalin based nanofibers, whilst the cyclohexane nanofibers exhibit a tensile strength of 18.2 ± 1.1 MPa with 46.7% ± 1.2% of elongation and a Young modulus of 39.1 ± 1.4 MPa [94]. The abovementioned results were obtained from bundles of nanofibers rather than individual fibers, these properties are strongly dependent on fiber orientation within the tested sample, bonding between fibers, and slip of one fiber over another [94].

Regarding nanofiber availability, there are several methods to prepare nanofiber systems. These methods include wet chemistry, Electrospinning (ES) [95] and Forcespinning^®^ (FS) [96] techniques. Most of the available literature has used ES processes; these studies have proven the potential of these nanofiber systems towards solving many of the challenges encountered in TE. ES processes have been limited to laboratory-based research given the challenges associated with increasing yield and opportunity to work with melt based systems. FS, a technique that has been recently introduced is based on developing nanofibers through the application of centrifugal forces. The method has been proven effective to produce yields that could satisfy industry requirements (i.e., several hundred meters per minute) as well as to produce nanofibers from melt based systems therefore removing the requirement of a solvent and subsequently the potential contamination of the materials with toxic organic solvents, and cost associated with the solvent itself and solvent recovery procedures. Other scaffolds had been produced by 3D printing procedures. Such biomimetic scaffolds are promising techniques as they could allow precise control over the geometry and microstructure [46,97].

Table 7 presents a summary of recently published work regarding the manufacture of nanofiber based surgical meshes.

Nanofiber systems are certainly showing a strong potential to be used in the next generation of surgical meshes, the increased availability (FS process) will certainly promote the development of practical applications. Nanofiber developed through the FS system have shown promising results regarding adhesion, growth, metabolic activity, proliferation, and viability of 3T3 cells [70,102]. It is expected that these systems will be used in combination with existent commercial meshes to satisfy other requirements such as mechanical strength needed to bear the intra-abdominal pressure exerted by human body and implantation requirements to mention some. Future studies in this area will include the effect of nanofiber morphology, mesh design (i.e., uniaxial aligned, radially aligned, orthogonally patterned) needed to improve structural properties, and in vivo testing.

In summary, this review synergistically complements recent reviews made in this important area. Table 8 presents a comparative table with recent published reviews [38,103,104,105,106]. Besides having in common the history and present scenario, this review also presents information regarding manufacturing methods (manufacturing of these meshes has a strong influence in the medical results, therefore the ultimate functionality will be strongly dependent upon the manufacturing method) and future perspectives.

## 5. Conclusions

Surgical meshes have become the system of choice for hernia repair. Even though it is not the optimum method, so far it is the one that has shown a lower rate of recurrence. Currently, there are more than 70 types of meshes commercially available. These are constructed from synthetic materials (absorbable, non-absorbable, or a combination of both) and animal tissue. Despite reducing rates of recurrence, hernia repair with surgical meshes still faces adverse effects such as infection, adhesion, and bowel obstruction. Most of these drawbacks are related to the chemical and structural nature of the mesh itself.

An optimum integration with the abdominal wall and negligible adhesion on the visceral side are the most important after sought features for the “ideal” mesh. A surgical mesh will trigger one of three different responses from the body: it may be integrated, encapsulated or degraded. In order to have a minimal inflammatory response to better integrate it to the body, it is highly important to improve biocompatibility.

To overcome this obstacle, researchers are actively exploring methods to improve biocompatibility, with the goal of developing a mesh that can be effectively incorporated with minimal inflammation and/or infection. Nanofibers have been recently considered as a strong potential intermediary structure to be used as a coating, given their ultralightweight quality, which could contribute to minimize the inflammatory response from the body and given its functional porosity, which could promote cell adhesion and proliferation.

## Figures and Tables

**Table 1:** Classification of commercially available first generation surgical meshes [38].

Product (Manufacturer)	Material	Pore Size (mm)	Absorbable	Weight (g/m^2^)	Filament	Mechanical Properties	Advantages and Disadvantages
Vicryl (Ethicon)	Polyglactin	0.4	Yes, fully (60–90 days)	56	Multifilament	Tensile strength of 78.2 ± 10.5 N/cm in longitudinal direction and 45.5 ± 13.5 N/cm in transverse direction.	Eliminates the risk of infectious disease transmission. Usually results in hernia recurrence after complete absorption
Dexon (Syneture)	Polyglycolic acid	0.75	Yes, fully (60–90 days)	56	Multifilament	N.A.	Adhesions fade as the mesh is absorbed. It is controversial whether the fibrous ingrowth into the prosthesis is sufficient to accomplish a permanent repair.
Sefil (B-Baun)	Polyglycolic acid	0.75	Yes, fully (60–90 days)	56	Multifilament	N.A.	High anatomic adaptability and low risk of late secondary infection. Retain 50% of its strength for 20 days.
Marlex (BARD)	PP	0.8	No	80–100	Monofilament	Tensile strength of 58.8 N/cm	High tensile strength. Evokes a chronic inflammatory reaction.
3D Max (BARD)	PP	0.8	No	80–100	Monofilament	Tensile strength of 124.7 N/cm	Anatomically designed. Reduced patient pain. Adhesions risk.
Polysoft (BARD)	PP	0.8	No	80–100	Multifilament	Burst strength of 558 N and a stiffness of 52.9 N/cm	Low infection risk. Not used in extraperitoneal spaces as produce dense adhesions *.
Prolene (Ethicon)	PP	0.8	No	80–100	Monofilament	Tensile strength of 156.5 N/cm	Facilitates fibrovascular ingrowth, infection resistance and improve compliance. Adhesions risk.
Surgipro (Autosuture)	PP	0.8	No	80–100	Multifilament	Tensile strength of 41.8 N/cm in longitudinal direction and 52.9 N/cm in transverse direction	High tensile strength, ease of handling and position and retains properties in vivo. Difficult complete wound healing caused by mesh structure.
Prolite (Atrium)	PP	0.8	No	80–100	Monofilament	Tensile strength of 138 N/cm	Monofilaments aligned in parallel spaced angles to maximizing material flexibility in two dimensions and a smooth and very uniform open architecture. Adhesions risk.
Trelex (Meadox)	PP	0.8	No	80–100	Multifilament	N.A.	*
Atrium (Atrium)	PP	0.8	No	80–100	Monofilament	Tensile strength of 56.2 N/cm	High tolerance to infection. Adhesions risk.
Premilene (B-Braun)	PP	0.8	No	80–100	Monofilament	Tensile strength of 41.4 N/cm in longitudinal direction and 36.5 N/cm in transverse direction	Mesh adaptation to the longitudinal and latitudinal axes of the connective tissue where is used for the reinforcement, rapid healing and tissue penetration. Adhesions risk.
Serapren (smooth)	PP	0.8	No	80–100	Multifilament	N.A.	*
Parietene (Covidien)	PP	0.8	No	80–100	Multifilament	Tensile strength of 38.9 ± 5.2 N/cm in longitudinal direction and 26.6 ± 4.2 N/cm in transverse direction	*
Prolene Light (Covidien)	PP	1.0–3.6	No	36–48	Monofilament	Tensile strength of 20 N/cm	Greater flexibility. Not used in intraperitoneal spaces as produce dense adhesions.
Optilene (B-Baun)	PP	1.0–3.6	No	36–48	Monofilament	Tensile strength of 58 N/cm	Soft, thin and pliable. Ideal for inguinal hernia repair to reduce chronic pain. Not used in extraperitoneal spaces as produce dense adhesions.
Mersilene (Ethicon)	POL	1.0–2.0	No	40	Multifilament	Tensile strength of 19 N/cm	Low infection risk. Evokes an aggressive macrophage and giant cell rich inflammatory reaction, followed by a dense fibrous ingrowth.
Goretex (Gore)	e-PTFE	0.003	No	Heavyweight	Multifilament	Minimum tensile strength of 16 N/cm	Smooth and strong. Evokes a chronic inflammatory reaction.

PP: Polypropylene. POL: Polyester. e-PTFE: Expanded polytetrafluoroethylene. N.A, Information not available in literature. * Duplicated properties.

**Table 2:** Classification of commercially available second generation surgical meshes [38].

Product (Manufacturer)	Material	Pore Size (mm)	Absorbable	Weight (g/m^2^)	Filament	Mechanical Properties	Advantages and Disadvantages
Vypro, Vypro II (Ethicon)	PP/polyglactin 910	>3	Partially (42 days)	25 & 30	Multifilament	Tensile strength of 16 N/cm	Significantly decreased rates of chronic pain. Higher rate of hernia recurrence.
Gore-Tex Dual Mesh Dual Mesh Plus (Gore)	e-PTFE	0.003–0.022	No	Heavyweight	Multifilament	Minimum tensile strength of 16 N/cm (Gore-Tex Dual Mesh) and 157.7 N/cm (Dual Mesh Plus)	Promotes host tissue growth and reduces tissue attachment. Infection risk.
Parietex (Covidien)	POL/collagen	>3	Partially (20 days)	75	Multifilament	Elasticity of 3.5 at 16 N	Short-term benefit for anti-adhesion property. Greater infection rate (57%).
Composix EX Dulex (BARD)	PP/e-PTFE	0.8	No	Lightweight	Monofilament	N.A.	Minimizes adhesions and provides optimal tissue ingrowth. Infection risk.
Proceed (Ethicon)	PP/cellulose	Large	Partially (<30 days)	45	Monofilament	Tensile strength of 56.6 N/cm	Low rates of hernia recurrence (3.7%). Risk of formation of visceral adhesions.
DynaMesh IPOM (FEG Textiltechnik)	PP/PVDF	1–2	Partially	60	Monofilament	Tensile strength of 11.1 ± 6.4 N/cm in longitudinal direction and 46.9 ± 9.7 N/cm in transverse direction	Minimal foreign body reaction. Adhesions risk.
Sepramesh (Genzyme)	PP/sodium	1–2	Partially (<30 days)	102	Monofilament	N.A.	Reduces adhesions and the optimal tissue ingrowth is promoted. Sticky consistency difficult the surgeon manipulation.
Ultrapro (Ethicon)	PP/PGC-25	>3	Partially (<140 days)	28	Monofilament	Tensile strength of 55 N/cm	Reduced inflammatory response. Adhesions risk.
Ti-Mesh (GfE)	PP/titanium	>1	No	16 & 35	Monofilament	Tensile strength of 12 N/cm (mesh of 16 g/m^2^) and 47 N/cm (mesh of 35 g/m^2^)	Reduced inflammatory response. Low tensile strength.
C-Qur (Atrium)	PP/omega 3	>1	Partially (120 days)	50	Monofilament	Ball burst strength of 170 ± 20.1 N	Short-term benefit for anti-adhesion property. No significant difference for adhesion grade or amount relative to other meshes.

PP: Polypropylene. e-PTFE: Expanded polytetrafluoroethylene. POL: Polyester. PVDF: Polyvinylidene fluoride. PGC-25: poliglecaprone 25. N.A, Information not available in literature.

**Table 3:** Classification of commercially available third generation surgical meshes [38].

Product (Manufacturer)	Material	Tensile Strength (MPa)	Advantages	Disadvantages
Surgisis (Cook)	Porcine (small intestine submucosa)	4	No refrigeration is required. Long history of safety data.	Requires hydration. Susceptible to collagenases.
FlexHD (J&J)	Human (acellular dermis)	10	No refrigeration or rehydration is required.	N.A.
AlloMax (Davol)	Human (acellular dermis)	23	No refrigeration or rehydration is required. Available in large sizes.	Hydration required.
CollaMend (Davol)	Porcine/Bovine (xenogenic acellular dermis)	11	No refrigeration or rehydration is required. Available in large sizes.	N.A.
Strattice (LifeCell)	Porcine/Bovine (xenogenic acellular dermis)	18	Available in large sheets.	Limited long-term follow up.
Permacol (Covidien)	Porcine/Bovine (xenogenic acellular dermis)	39	No refrigeration or rehydration is required. Available in large sizes.	N.A.
XenMatrix (Davol)	Porcine/Bovine (xenogenic acellular dermis)	14	Available in large sheets.	Limited long-term follow up.

N.A. Information not available in literature.

**Table 4:** Classification of commercially available surgical meshes [59].

Mesh	Structural Textile Technique	Polymer	Fiber
Marlex	Woven	PP	Mono
Prolene^®^	Warp	PP	Mono
Atrium^®^	Warp	PP	Mono
Vypro^®^	Warp	PP/PG-910	Multi
UltraPro^®^	Warp	PP/PGC-25	Mono
TiMesh^®^	Warp	PP/Ti	Mono
DualMesh^®^	Warp	e-PTFE	Foil *
Mersilene^®^	Warp	Polyethylene Terephthalate (PET)	Multi
Dynamesh^®^	Warp	PVDF	Mono
Vycril^®^	Woven	Resorbable undyed Polyglactin	Multi
Gore-Tex^®^	Woven	e-PTFE	Multi

* Membrane/patch.

**Table 5:** Material properties of surgical mesh coatings.

PLLA/PGA	ORC/SCMC	NVP/BMA	Omega-3 Fatty Acid	RMSC/HDF/RKF	Collagen/Chitosan	NCSP	Titanium
Variable degradation rate	Reduce mesh adhesions	Reduce mesh adhesions	Minimal risk of mesh contraction	Affinity towards fibroblasts	Weak tensile properties	Anti-inflammatory	Provides mechanical integrity
Hydrophilicity	Absorbable	Hydrophilicity	Absorbable	Favourable cell adhesion	Negligible effect on biomechanical properties	Antimicrobial	Non-absorbable

PLLA: Polylactic acid. PGA: Polyglycolic acid. ORC: Oxygenated regenerated cellulose. SCMC: Carboxymethylcellulose. NVP: N-vinyl pyrrolydone. BMA: N-butylmethacrylate. RMSC: Messenchymal stem cells. HDF: Human dermal. RKF: Rat kidney fibroblasts. NCSP: Nanocrystalline silver particles.

**Table 6:** Examples of surgical mesh coating parameters.

Reference	Analyzed Parameter	
	Material	Surface Density
Pascual et al. [86]	Oxidized collagen Chitosan	Oxidized collagen 95%/ Chitosan 5%
Ciechańska et al. [71]	MBC	6.7 g/m^2^ (one side) 5.31 g/m^2^ (two sides)
Cohen et al. [81]	NCSP	310 g/m^2^ 640 g/m^2^ 1130 g/m^2^
Niekraszewics et al. [85]	Chitosan	20 g/m^2^ (one side) 20 g/m^2^ (two sides)

MBC: Modified bacterial cellulose. NCSP: Nanocrystalline silver particles.

**Table 7:** Nanofiber based surgical meshes.

Nanofiber Material	Manufacturing Process	Diameter (nm)	Tensile Strength (MPa)	Advantages and Disadvantages	Reference
Poly-ε-caprolactone (PCL)	Electrospinning	1280 ± 330	3.11 ± 1.09	Better adhesion, growth, metabolic activity, proliferation and viability of 3T3 Fibroblasts. Lack of in vivo testing.	[87,98]
Polydioxanone (PDO)	Electrospinning	860 ± 420	3.76 ± 0.49	Bioresorbable polymer. Reduction of long-term foreign body response (LTFBR). No fulfill the mechanical requirements.	[99]
Polylactide-Co-Glycolide (PLGA 8218)	Electrospinning	3280 ± 570	6.47 ± 0.41	Exceed the minimum mechanical requirements for hernia repair applications. Bioresorbable polymer. Reduction of LTFBR. Lack of in vivo testing.	
PLLA	Electrospinning	1480 ± 670	3.59 ± 0.25	In vivo advantages. Exceed the minimum mechanical requirements for hernia repair applications. Lack of in vivo testing.	
Polyurethane (PU)	Electrospinning	890 ± 330	18.9 ± 5.9	Elastic deformation.	
PET	Electrospinning	710 ± 280	3.17 ± 0.23	Adequate mechanical attributes. No evidence of intestinal adhesions. Trigger of a large foreign body reaction.	[100]
PET/Chitosan	Electrospinning	3010 ± 720	2.89 ± 0.27	Adequate mechanical attributes. No evidence of intestinal adhesions. Trigger of a large foreign body reaction.	
PCL/Collagen	Electrospinning	1000	2.13 ± 0.36	Biological and biomechanical stable, support skeletal muscle cell ingrowth and neo-tissue formation	[101]

PCL: Poly-ε-caprolactone. PDO: Polydioxanone. PLGA 8218: Polylactide-Co-Glycolide. PU: Polyurethane. PET: Polyethylene terephthalate.

**Table 8:** Aspects related to hernia meshes compared in recently published reviews.

	Baylon et al. (This Review)	Brown et al. [38]	Sanbhal et al. [103]	Guillaume et al. [104]	Todros et al. [105]	Todros et al. [106]
Introduction	√	√	√	√	√	√
History	√	√	-	-	-	-
Present Scenario	√	√	√	√	√	√
Properties Discussed	Elasticity/tensile strength Pore Size Weight (density) Constitution Material absorption	Tensile strength Pore Size Weight Reactivity/Biocompatibility Elasticity Constitution Shrinkage Complications	Weight Pore Shape, size/porosity Mesh elasticity/strength	Properties discussed for particular meshes, varies from the type of mesh being discussed.	Pore size Density thickness	Biomechanical properties Uniaxial tensile testing Biaxial tensile testing Ball burst testing
Surgical Mesh	√	√	√	√	√	√
Manufacturing Processes	> 2 processes considered	-	-	-	-	-
Future Perspectives	2 perspectives considered	-	√	√	-	-
Comments	Comparison of meshes divided by generations: First generation (18 meshes), second generation, (10 meshes), third generation (7 meshes)	Comparison of meshes divided by constitution, Multi (3 meshes), multifilament and monofilament (13 meshes), and foil (1 mesh). Biomaterial meshes (10 meshes)	Comparison between synthetic meshes (15 meshes) Comparison between composite meshes (12 meshes)	Meshes divided by Biologically Derived Matrices, Biodegradable synthetic structures, Anti-inflammatory mesh, Meshes with enhanced cytocompatibility, Anti-adhesive Mesh, Antibacterial meshes. Review also discusses mesh fixation, self-expanding systems, post-implantation visible mesh, cell coated meshes, and growth factor loaded meshes.	Comparison between synthetic surgical meshes: HWPP (5 meshes), LWPP (6 meshes), PET (1mesh), ePTFE (1 mesh), PVDF (1 mesh) Comparison between Multilayered meshes (10 meshes)	Comparison between synthetic surgical meshes: HWPP (5 meshes), LWPP (3 meshes), PET (1 mesh), ePTFE (1 mesh), PVDF (1 mesh). Comparison between Multilayered Meshes (10 meshes)
Total meshes compared	35	27	27	-	24	21

## References

[1] Williams L.S., Hopper P.D. (2015). Understanding Medical-Surgical Nursing.

[2] Dabbas N., Adams K., Pearson K., Royle G.T. (2011). Frequency of abdominal wall hernias: Is classical teaching out of date?. J. R Soc. Med. Short Rep..

[3] Bendavid R., Abrahamson J., Arregui M.E., Flament J.B., Phillips E.H. (2001). Abdominal Wall Hernias: Principles and Management.

[4] Heniford B.T. (2015). Hernia Handbook.

[5] Kingsnorth A. (2004). Treating inguinal hernias: Open mesh Lichtenstein operation is preferred over laparoscopy. BMJ.

[6] Li X., Kruger J.A., Jor J.W., Wong V., Dietz H.P., Nash M.P., Nielsen P.M. (2014). Characterizing the ex vivo mechanical properties of synthetic polypropylene surgical mesh. J. Mech. Behav. Biomed. Mater..

[7] CORDIS: Community Research and Development Information Service. http://cordis.europa.eu/result/rcn/178015_en.html.

[8] Bard Davol Inc.. https://www.davol.com/index.cfm/_api/render/file/?method=inline&fileID=90027245-5056-9046-9529B0C67424C711.

[9] Pandit A.S., Henry J.A. (2004). Design of surgical meshes—An engineering perspective. Technol. Heal. Care.

[10] Melero Correas H. (2008). Caracterización Mecánica de Mallas Quirúrgicas Para la Reparación de Hernias Abdominales. Master Thesis.

[11] Zhu L.-M., Schuster P., Klinge U. (2015). Mesh implants: An overview of crucial mesh parameters. World J. Gastrointest. Surg..

[12] Billroth T., Welch W.H. (1924). The Medical Sciences in the German Universities: A Study in the History of Civilization.

[13] Chowbey P. (2012). Endoscopic Repair of Abdominal Wall Hernias.

[14] Greenberg J.A., Clark R.M. (2009). Advances in suture material for obstetric and gynecologic surgery. Rev. Obstet. Gynecol..

[15] LeBlanc K.A. (2003). Laparoscopic Hernia Surgery an Operative Guide.

[16] Usher F.C., Fries J.G., Ochsner J.L., Tuttle L.L. (1959). Marlex mesh, a new plastic mesh for replacing tissue defects. II. A new plastic mesh for replacing tissue defects. AMA Arch. Surg..

[17] Usher F.C., Hill J.R., Ochsner J.L. (1959). Hernia repair with Marlex mesh. A comparison of techniques. Surgery.

[18] Klinge U., Klosterhalfen B., Birkenhauer V., Junge K., Conze J., Schumpelick V.J. (2002). Impact of polymer pore size on the interface scar formation in a rat model. Surg. Res..

[19] EU Hernia Trialists Collaboration (2002). Repair of groin hernia with synthetic mesh: Meta-analysis of randomized. Ann. Surg..

[20] Stowe J.A. (2015). Development and Fabrication of Novel Woven Meshes as Bone Graft Substitutes for Critical Sized Defects. Ph.D. Thesis.

[21] Hawn M.T., Gray S.H., Snyder C.W., Graham L.A., Finan K.R., Vick C.C. (2011). Predictors of mesh explantation after incisional hernia repair. Am. J. Surg..

[22] Carbajo M.A., Martín del Olmo J.C., Blanco J.I., De la Cuesta C., Toledano M., Martín F., Vaquero C., Inglada L. (1999). Laparoscopic treatment vs open surgery in the solution of major incisional and abdominal wall hernias with mesh. Surg. Endosc..

[23] Schumpelick V., Fitzgibbons R.J. (2010). Hernia Repair Sequelae.

[24] Bendavid R. (1994). Prostheses and Abdominal Wall Hernias.

[25] Zogbi L., Pignatello R. (2008). The Use of Biomaterials to Treat Abdominal Hernias. Biomaterials Applications for Nanomedicine.

[26] Anderson J.M. (2001). Biological Response to Materials. Annu. Rev. Mater. Res..

[27] Batchelor A.W., Chandrasekaran M. (2004). Service Characteristics of Biomedical Materials and Implants.

[28] Santambrogio L. (2015). Biomaterials in Regenerative Medicine and the Immune System.

[29] Acevedo A. (2008). Mallas sintéticas Irreabsorbibles su desarrollo en la cirugía de las hernias abdominals. Revista Chilena Cirugía.

[30] Tang L., Ugarova T.P., Plow E.F., Eaton J.W. (1996). Molecular determinates of acute inflammatory response to biomaterials. J. Clin. Invest..

[31] Busuttil S.J., Ploplis V.A., Castellino F.J., Tang L., Eaton J.W., Plow E.F. (2004). A central role for plasminogen in the inflammatory response to biomaterials. J. Thromb. Haemost..

[32] Earle D.B., Mark L.A. (2008). Prosthetic Material in Inguinal Hernia Repair: How Do I Choose?. Surg. Clin. North Am..

[33] Schaechter M. (2009). Encyclopedia of Microbiology.

[34] Jacob B.P., Ramshaw B. (2013). The SAGES Manual of Hernia Repair.

[35] Ramshaw B., Bachman S. (2007). Surgical materials for ventral hernia repair. Gen. Surg. News.

[36] Anderson J.M., Rodriguez A., Chang D.T. (2008). Foreign Body Reaction to Biomaterials. Semin. Immunol..

[37] Chu C.-C., von Fraunhofer J.A., Greisler H.P. (1997). Wound Closure Biomaterials and Devices.

[38] Brown C.N., Finch J.G. (2010). Which mesh for hernia repair?. Ann. R. Coll. Surg. Engl..

[39] Klinge U., Klosterhalfen B., Schumpelick V. (1999). Foreign Body Reaction to Meshes of Used for the Repair of Abdominal Wall Hernias. Eur. J. Surg..

[40] Junge K., Klinge U., Prescher A., Giboni P., Niewiera M., Schumpelick V. (2001). Elasticity of the anterior abdominal wall and impact for reparation of incisional hernias using mesh implants. Hernia.

[41] Pourdeyhimi B.J. (1989). Porosity of surgical mesh fabrics: New technology. Biomed. Mater. Res..

[42] Bilsel Y., Abci I. (2012). The search for ideal hernia repair; mesh materials and types. Int. J. Surg..

[43] Winters J.C., Fitzgerald M.P., Barber M.D. (2006). The use of systhetic mesh in female pelvic reconstructive surgery. BJU Int..

[44] Halm J.A. (2005). Experimental and Clinical Approaches to Hernia Treatment and Prevention. Ph.D. Thesis.

[45] Cortes R.A., Miranda E., Lee H., Gertner M.E., Norton J., Barie P.S., Bollinger R.R., Chang A.E., Lowry S., Mulvihill S.J., Pass H.I., Thompson R.W. (2008). Biomaterials and the evolution of hernia repair II: Composite meshes. Surgery.

[46] Tamayol A., Akbari M., Annabi N., Paul A., Khademhosseini A., Juncker D. (2013). Fiber-based tissue engineering: Progress, challenges, and opportunities. Biotechnol. Adv..

[47] Blair T. (2015). Biomedical Textiles for Orthopaedic and Surgical Applications: Fundamentals, Applications and Tissue Engineering.

[48] King M.W., Gupta B.S., Guidoin R. (2013). Biotextiles as Medical Implants.

[49] Listner G. (1971). Polypropylene Monofilament Sutures. U.S. Patent.

[50] Hutton J.D., Dumican B.L. (2001). Braided Polyester Suture and Implantable Medical Device. U.S. Patent.

[51] Gore R.W. (1976). Process for Producing Porous Products. U.S. Patent.

[52] Pott P.P., Schwarz M.L.R., Gundling R., Nowak K., Hohenberger P., Roessner E.D. (2012). Mechanical properties of mesh materials used for hernia repair and soft tissue augmentation. PLoS ONE.

[53] Lennard D.J., Menezes E.V., Lilenfeld R. (1990). Pliabilized Polypropylene Surgical Filaments. U.S. Patent.

[54] Laurencin C.T., Nair L.S., Bhattacharyya S., Allcock H.R., Bender J.D., Brown P.W., Greish Y.E. (2007). Polymeric Nanofibers for Tissue Engineering and Drug Delivery. U.S. Patent.

[55] Zhukovsky V., Rovinskaya L., Vinokurova T., Zhukovskaya I. (2002). The Development and Manufacture of Polymeric Endoprosthetic Meshes for the Surgery of Soft Tissues. Autex Res. J..

[56] Rousseau R.A., Dougherty R. (2003). Knitted Surgical Mesh. U.S. Patent.

[57] Schumpelick V., Nyhus L. (2004). Meshes: Benefits and Risks.

[58] Cobb W.S., Peindl R.M., Zerey M., Carbonell A.M., Heniford B.T. (2009). Mesh terminology 101. Hernia.

[59] Klosterhalfen B., Junge K., Klinge U. (2005). The lightweight and large porous mesh concept for hernia repair. Expert Rev. Med. Devices.

[60] Wang X., Han C., Hu X., Sun H., You C., Gao C., Haiyang Y. (2011). Applications of knitted mesh fabrication techniques to scaffolds for tissue engineering and regenerative medicine. J. Mech. Behav. Biomed. Mater..

[61] Camp Tibbals E., Leinsing K.R., DeMarco P.B. (2000). Flat-Bed Knitting Machine and Method of Knitting. U.S. Patent.

[62] Dougherty R., Vishvaroop A. (2006). Surgical Tricot. U.S. Patent.

[63] Ting H. (2011). A Study of Three Dimensional Warp Knits for Novel Applications as Tissue Engineering Scaffolds. Master Thesis.

[64] Spencer D.J. (1983). Knitting Technology: A Comprehensive Handbook and Practical Guide to Modern Day Principles and Practices.

[65] Deichmann T., Michaelis I., Junge K., Tur M., Michaeli W., Gries T. (2009). Textile Composite Materials for Small Intestine Replacement. Autex Res. J..

[66] Raz S. (1987). Warp Knitting Production.

[67] Emans P.J., Schreinemacher M.H., Gijbels M.J., Beets G.L., Greve J.W., Koole L.H., Bouvy N.D. (2009). Polypropylene Meshes to Prevent Abdominal Herniation: Can Stable Coatings Prevent Adhesions in the Long Term?. Ann. Biomed. Eng..

[68] Van’t Riet M., de Vos van Steenwijk P.J., Bonthuis F., Marquet R.L., Steyerberg E.W., Jeekel J., Bonjer H.J. (2003). Prevention of Adhesion to Prosthetic Mesh: Comparison of Different Barriers Using an Incisional Hernia Model. Ann. Surg..

[69] Ebersole G.C., Buettmann E.G., MacEwan M.R., Tang M.E., Frisella M.M., Matthews B.D., Deeken C.R. (2012). Development of novel electrospun absorbable polycaprolactone (PCL) scaffolds for hernia repair applications. Surg. Endosc. Other Interv. Tech..

[70] Xu F., Weng B., Gilkerson R., Materon L.A., Lozano K. (2015). Development of tannic acid/chitosan/pullulan composite nanofibers from aqueous solution for potential applications as wound dressing. Carbohydr. Polym..

[71] Ciechańska D., Kazimierczak J., Wietecha J., Rom M. (2012). Surface Biomodification of Surgical Meshes Intended for Hernia Repair. Fibres Text. East. Eur..

[72] Karamuk Z.E. (2001). Embroidered Textiles for Medical Applications: New Design Criteria with Respect to Structural Biocompatibility. Ph.D. Thesis.

[73] Norton J.A., Barie P.S., Bollinger R.R., Chang A.E., Lowry S.F., Mulvihill S.J., Pass H.I., Thompson R.W. (2008). Surgery.

[74] Yelimlieş B., Alponat A., Cubukçu A., Kuru M., Oz S., Erçin C., Gönüllü N. (2003). Carboxymethylcellulose coated on visceral face of polypropylene mesh prevents adhesion without impairing wound healing in incisional hernia model in rats. Hernia.

[75] Franklin M.E., Voeller G., Matthews B.D., Earle D.B. (2010). The Benefits of Omega-3 Fatty Acid-Coated Mesh in Ventral Hernia Repair. Spec. Rep..

[76] Gao Y., Liu L.J., Blatnik J.A., Krpata D.M., Anderson J.M., Criss C.N., Posielski N., Novitsky Y.W. (2014). Methodology of fibroblast and mesenchymal stem cell coating of surgical meshes: A pilot analysis. J. Biomed. Mater. Res. B. Appl. Biomater..

[77] Kidoaki S., Kwon I.K., Matsuda T. (2005). Mesoscopic spatial designs of nano- and microfiber meshes for tissue-engineering matrix and scaffold based on newly devised multilayering and mixing electrospinning techniques. Biomaterials.

[78] Lamber B., Grossi J.V., Manna B.B., Montes J.H., Bigolin A.V., Cavazzola L.T. (2013). May polyester with collagen coating mesh decrease the rate of intraperitoneal adhesions in incisional hernia repair?. Arq. Bras. Cir. Dig..

[79] Van’t Riet M., Burger J.W., Bonthuis F., Jeekel J., Bonjer H.J. (2004). Prevention of adhesion formation to polypropylene mesh by collagen coating: A randomized controlled study in a rat model of ventral hernia repair. Surg. Endosc..

[80] Niekraszewicz A., Kucharska M., Wawro D., Struszczyk M.H., Kopias K., Rogaczewska A. (2007). Development of a Manufacturing Method for Surgical Meshes Modified by Chitosan. Fibres Text. East. Eur..

[81] Cohen M.S., Stern J.M., Vanni A.J., Kelley R.S., Baumgart E., Field D., Libertino J.A., Summerhayes I.C. (2007). In Vitro Analysis of a Nanocrystalline Silver-Coated Surgical Mesh. Surg. Infect. (Larchmt).

[82] Junge K., Rosch R., Klinge U., Saklak M., Klosterhalfen B., Peiper C., Schumpelick V. (2005). Titanium coating of a polypropylene mesh for hernia repair: Effect on biocompatibility. Hernia.

[83] Scheidbach H., Tannapfel A., Schmidt U., Lippert H., Köckerling F. (2004). Influence of Titanium Coating on the Biocompatibility of a Heavyweight Polypropylene Mesh. Eur. Surg. Res..

[84] Niekraszewicz A., Kucharska M., Wawro D., Struszczyk M.H., Rogaczewska A. (2007). Partially Resorbable Hernia Meshes. Prog. Chem. Appl. Chitin Its Deriv..

[85] Niekraszewicz A., Kucharska M., Struszczyk M.H., Rogaczewska A., Struszczyk K. (2008). Investigation into Biological, Composite Surgical Meshes. Fibres Text. East. Eur..

[86] Pascual G., Sotomayor S., Rodríguez M., Bayon Y., Bellón J.M. (2013). Behaviour of a New Composite Mesh for the Repair of Full-Thickness Abdominal Wall Defects in a Rabbit Model. PLoS ONE.

[87] Plencner M., East B., Tonar Z., Otáhal M., Prosecká E., Rampichová M., Krejčí T., Litvinec A., Buzgo M., Míčková A., Nečas A. (2014). Abdominal closure reinforment by using polypropylene mesh functionalized with poly-ε-caprolactone nanofibers and growth factors for prevention of incisional hernia formation. Int. J. Nanomedicine.

[88] Alves da Silva M.L., Martins A., Costa-Pinto A.R., Costa P., Faria S., Gomes M., Reis R.L., Neves N.M. (2010). Cartilage Tissue Engineering using electrospun PCL nanofiber meshes and MSCs. Biomacromolecules.

[89] Popat K. (2010). Nanotechnology in Tissue Engineering and Regenerative Medicine.

[90] Vasita R., Katti D.S. (2006). Nanofibers and their applications in tissue engineering. Int. J. Nanomedicine.

[91] Dorband G.C., Liland A., Menezes E., Steinheuser P., Popadiuk N.M., Failla S.J. (1987). Surgical Fastening Device and Method for Manufacture. U.S. Patent.

[92] Brown P., Stevens K. (2007). Nanofibers and Nanotechnology in Textiles.

[93] Watanabe K., Kim B.S., Kim I.S. (2011). Development of Polypropylene Nanofiber Production System. Polym. Rev..

[94] Watanabe K., Nakamura T., Kim B.S., Kim I.S. (2011). Effect of organic solvent on morphology and mechanical properties of electrospun syndiotactic polypropylene nanofibers. Polym. Bull.

[95] Huang Z.-M., Zhang Y.Z., Kotaki M., Ramakrishna S. (2003). A review on polymer nanofibers by electrospinning and their applications in nanocomposites. Compos. Sci. Technol..

[96] Padron S., Fuentes A., Caruntu D., Lozano K. (2013). Experimental study of nanofiber production through forcespinning. J. Appl. Phys..

[97] Yarlagadda P., Chandrasekharan M., Shyan J.Y. (2005). Recent Advances and Current Developments in Tissue Scaffolding. Biomed. Mater..

[98] Plencner M., Prosecká E., Rampichová M., East B., Buzgo M., Vysloužilová L., Hoch J., Amler E. (2015). Significant improvement of biocompatibility of polypropylene mesh for incisional hernia repair by using poly-ε-caprolactone nanofibers functionalized with thrombocyte-rich solution. Int. J. Nanomedicine.

[99] Chakroff J., Kayuha D., Henderson M., Johnson J. (2015). Development and Characterization of Novel Electrospun Meshes for Hernia Repair. Int. J. Nanomedicine.

[100] Veleirinho B., Coelho D.S., Dias P.F., Maraschin M., Pinto R., Cargnin-Ferreira E., Peixoto A., Souza J.A., Ribeiro-do-Valle R.M., Lopes-da-Silva J.A. (2014). Foreign Body Reaction Associated with PET and PET/Chitosan Electrospun Nanofibrous Abdominal Meshes. PLoS ONE.

[101] Zhao W., Ju Y.M., Christ G., Atala A., Yoo J.J., Lee S.J. (2013). Diaphragmatic muscle reconstruction with an aligned electrospun poly(ε-caprolactone)/collagen hybrid scaffold. Biomaterials.

[102] Xu F., Weng B., Materon L.A., Gilkerson R., Lozano K. (2014). Large-scale production of ternary composite nanofiber membrane for wound dressing applications. J. Bioact. Compat. Polym. Biomed. Appl..

[103] Sanbhal N., Miao L., Xu R., Khatri A., Wang L. (2017). Physical structure and mechanical properties of knitted hernia mesh materials: A review. J. Ind. Text..

[104] Guillaume O., Teuschl A.H., Gruber-Blum S., Fortelny R.H., Redl H., Petter-Puchner A. (2015). Emerging trends in abdominal wall reinforcement: Bringing bio-functionality to meshes. Adv. Healthc. Mater..

[105] Todros S., Pavan P.G., Natali A.N. (2017). Synthetic surgical meshes used in abdominal wall surgery: Part I—Materials and structural conformation. J. Biomed. Mater. Res. Part B: Appl. Biomater..

[106] Todros S., Pavan P.G., Pachera P., Natali A.N. (2017). Synthetic surgical meshes used in abdominal wall surgery: Part II—Biomechanical aspects. J. Biomed. Mater. Res. Part B: Appl. Biomater..

